# Facile bioactive transformation of magnesium alloy surfaces for surgical implant applications

**DOI:** 10.3389/fbioe.2023.1156525

**Published:** 2023-08-01

**Authors:** Cheng-Chieh Wang, Jing-Ya Hung, Jun-Yen Uan, Chih-Yuan Fang, Yu-Lin Kuo, Wei-Jen Chang, Yoichi Ohiro, Ying-Sui Sun

**Affiliations:** ^1^ Division of Endodontics, Department of Dentistry, Taipei Medical University Hospital, Taipei, Taiwan; ^2^ School of Dental Technology, College of Oral Medicine, Taipei Medical University, Taipei, Taiwan; ^3^ Department of Materials Science and Engineering, National Chung Hsing University, Taichung, Taiwan; ^4^ Division of Oral and Maxillofacial Surgery, Department of Dentistry, Wan Fang Hospital, Taipei, Taiwan; ^5^ Department of Mechanical Engineering, National Taiwan University of Science and Technology, Taipei, Taiwan; ^6^ School of Dentistry, College of Oral Medicine, Taipei Medical University, Taipei, Taiwan; ^7^ Dental Department, Shuang-Ho Hospital, Taipei Medical University, Taipei, Taiwan; ^8^ Oral and Maxillofacial Surgery, Division of Oral Pathobiological Science, Faculty of Dental Medicine and Graduate School of Dental Medicine, Hokkaido University, Sapporo, Japan

**Keywords:** magnesium alloy, biodegradable, corrosion resistance, bone screw, osteoblasts responses

## Abstract

The market for orthopedic implant alloys has seen significant growth in recent years, and efforts to reduce the carbon footprint of medical treatment (i.e., green medicine) have prompted extensive research on biodegradable magnesium-based alloys. Magnesium alloys provide the mechanical strength and biocompatibility required of medical implants; however, they are highly prone to corrosion. In this study, Mg-9Li alloy was immersed in cell culture medium to simulate degradation in the human body, while monitoring the corresponding effects of the reaction products on cells. Variations in pH revealed the generation of hydroxyl groups, which led to cell death. At day-5 of the reaction, a coating of MgCO_3_ (H_2_O)_3_, HA, and α -TCP appeared on sample surfaces. The coating presented three-dimensional surface structures (at nanometer to submicron scales), anti-corrosion effects, and an altered surface micro-environment conducive to the adhesion of osteoblasts. This analysis based on bio-simulation immersion has important implications for the clinical use of Mg alloys to secure regenerated periodontal tissue.

## 1 Introduction

It is expected that for the next several years, low-carbon medical care will be a major target in the transition toward sustainable net-zero development. One approach to reducing the carbon emissions associated with medical care involves reducing the burden of additional treatment. To this end, researchers have been developing biodegradable metal alloys for the fabrication of so-called green medical devices. One recent development is referred to as temporary implants, which are commonly fabricated using alloys of magnesium, due to their excellent biocompatibility and Young’s modulus close to that of bone. Note however that magnesium alloy degrades too rapidly for most clinical applications other than as bone screws for fixation and suturing (Lu et al., 2021). In recent years, scholars have invested considerable time and cost seeking to extend the clinical applicability of these materials.

Mg is essential to the functioning of many human tissue types, and Mg ions are abundant in biological fluids and stored in large quantities in bone tissue. Mg is a cofactor for enzymes in human metabolism. Mg plays important roles in maintaining wall tension in blood vessels, promoting tissue healing, and regulating muscle contraction. It also participates in the formation of antibodies, tissue calcification, and other processes. Mg is easily corroded in the human body; however, Mg ions are not cytotoxic in a physiological environment and can be excreted in urine (Wolf and Cittadini, 2003). Mg is highly biocompatible, and the other elements used in most Mg alloys do not present a toxic hazard. Magnesium concentrations in serum/plasma generally remain within a healthy range; however, bone and soft tissue are prone to gradual depletion (Elin, 1987). Unlike other ions, magnesium ions maintain a transmembrane concentration gradient in cells. Normal intracellular and extracellular concentrations of free Mg^2+^ generally range from 10 to 30 mM (Rahman et al., 2020). In a previous study on the *in vivo* degradation of magnesium alloys (3% Al + 1% Zn, 9% Al + 1% Zn, 4% Y + 3% Nd, and 4% Li + 4% Al + 2% Ce), it was found that Mg corroded more slowly when alloyed with Li and Al. In that study, alloy elements were observed in the corrosion layer adjacent to the amorphous calcium phosphate layer but not in adjacent bone tissue (Witte, 2015).

Alloying with lithium (Li) can alter the hexagonal close packed (hcp) structure of Mg to form a body centered cubic (bcc) structure. Li is the metal with the lowest density, such that combining metallic Mg with Li results in an ultra-light alloy with outstanding properties. Binary alloys of Mg and Li are die-cast in the form of implants using the vacuum stirring casting method. Mg-Li alloys can be extruded to improve stretchability or to increase the content of secondary phases and thereby enhance hardness (Ebel et al., 1980). The biocompatibility of Li is reflected in its wide clinical use as a psychotropic drug. Several studies have also confirmed that Li promotes the osteogenic differentiation of bone marrow mesenchymal stem cells by activating the Wnt/GSK-3 signaling pathway, thereby promoting bone regeneration and ameliorating osteoporosis (Staiger et al., 2006). The fact that Li promotes osteogenesis has attracted considerable attention in the field of bone tissue engineering (Rahulan et al., 2019). However, the low corrosion resistance of Mg-Li alloy, due largely to the chemical reactivity of Li, limits its clinical applicability (Li et al., 2021; Chen et al., 2022). The primary disadvantage of all Mg alloys is rapid corrosion, which can lead to uncontrolled degradation, structural failure, hydrogen formation, and elevated pH levels. Note that the release of high concentrations of corrosion products can have detrimental effects on biocompatibility (Song et al., 2009). Nonetheless, the susceptibility of Mg-Li-based alloys to corrosion can be suppressed by subjecting implants to surface treatment prior to insertion.

Mg alloy implants have several advantages over other materials, including 1) biocompatibility and the promotion of osteogenesis, 2) biodegradability to eliminate the need for secondary surgery, 3) good mechanical properties, 4) processability and dimensional stability, and 5) high compression resistance (Elin, 1987). The Young’s modulus of human bone (3–20 GPa) is closer to that of Mg alloy (41–45 GPa) than to other implant materials, such as stainless steel (190–205 GPa), Ti (110–117 GPa), and Co-Cr alloys (230 GPa) (Rahman et al., 2020). Researchers have also developed numerous methods by which to tune the degradability of magnesium and its alloys.

Previous research has focused largely on magnesium-lithium-based alloys (Mg-9Li-1Zn) with mechanical strength comparable to that of natural bone. Researchers have developed several surface treatment methods to enhance the corrosion resistance of Mg alloys; however, most of those methods are complex or require expensive machinery (Toorani et al., 2019). Our primary objective in this research was to develop a simple surface treatment method involving the repeated immersion of alloy implants in solutions that simulate human physiological conditions (i.e., cell culture medium). We then assessed the suitability of the devices for implantation within the human body. Experiments were conducted to analyze the corrosion resistance of the material as well as the growth of bone cells on the surface of the material. Note that this testing did not involve analyzing the composition of surface deposits or the physical properties of the material after impregnation. Our analysis of biomimetic responses has important implications for the clinical application of Mg-Li-based alloys in the form of bone screws to promote the regeneration of periodontal ligaments without the need for subsequent surgical resection. We found that by controlling the rate of magnesium alloy degradation, it should be possible to create dental implants that meet clinical requirements. The proposed immersion method was shown to improve the corrosion resistance and bio-responsiveness of the material surface. The proposed approach to surface modification is simple to implement and consumes very little energy, compared to alloy recasting (Cao et al., 2008). Nonetheless, further studies will be required to elucidate the long-term cellular responses and implications for tissue regeneration.

## 2 Materials and methods

Mg-Li and LZ alloy samples were fabricated via melting and casting. As-cast samples underwent homogenization treatment at 330°C, followed by air cooling to room temperature. The samples were then hot rolled at 240°C to fabricate the Mg-Li and LZ alloy plates with a thickness of 0.8 mm. A water-jet cutter was used to cut the plates into discs for subsequent analysis.

### 2.1 Surface treatment of magnesium-lithium alloy

The mechanical properties of Mg can be greatly enhanced by alloying it with Li and/or Zn to form binary Mg-Li alloys or ternary Mg-Li-X (X = Al, Zn, Ca, Y, Ce) alloys (Peng et al., 2019). This study focused on a Li-containing Mg alloy (Mg-9Li-1Zn; hereafter referred to as LZ). Disc-shaped samples of this material measuring 2 cm in diameter with a thickness of 2 mm were fabricated.

**FIGURE 1:**
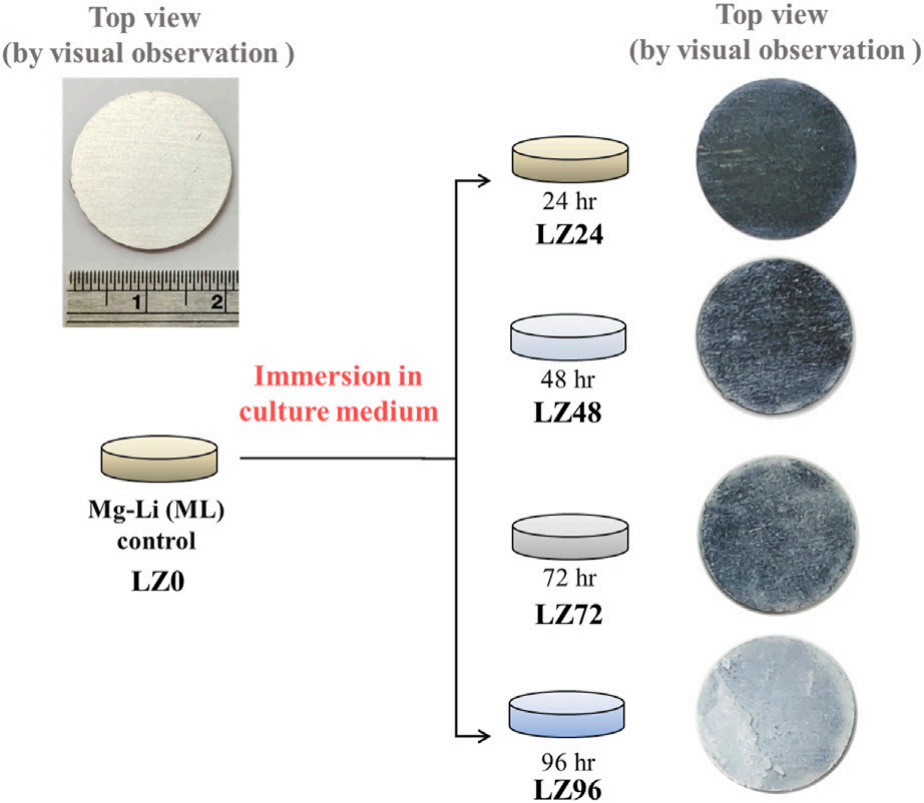
Schematic illustration showing sample preparation and a top-view of all samples.

The surface oxide layer of the LZ was removed using #1200 silicon carbide water sandpaper, after which the samples were dried and stored in a moisture-proof box until use. The LZ samples were then immersed in osteoblast culture medium to simulate degradation under *in vitro* environmental conditions (Panasonic MCO-18AC-PT). The surface properties of the material (physical, chemical, and corrosion resistance) and cell attachment were examined at various time points (0, 24, 48, 72, and 96 h) throughout the immersion process, as indicated by the naming of the samples (i.e., LZ0, LZ24, LZ48, LZ72, and LZ96). Samples of osteoblast culture medium were collected to test for ion release (see Figure 1).

### 2.2 Material surface properties

Top-view visual observation and field emission scanning electron microscopy (FE-SEM, JEOL JSM-6500F, Japan) were used to observe the surface morphology and cell adhesion morphology of the LZ samples. Energy-dispersive X-ray spectrometry (EDS, JEOL JSM-6500F, Japan) and X-ray diffraction (XRD, D8 DISCOVER - Bruker) were respectively used to identify surface element composition and chemical composition. Surface roughness was measured using a white-light interferometer (Filmetrics^®^ Profilm3D^®^ Optical Profilometer, KLA, United States).

### 2.3 Ion release

Inductively coupled plasma optical emission spectrometry (ICP-OES, Thermo Fisher Scientific iCAP TQ) was used to analyze the concentration of ions released into the culture medium as an indication of corrosion rate. We also measured the pH of the culture medium at 3 h to quantify the release of hydroxy groups associated with the corrosion process. Note that any change in the pH of cell culture medium could affect cell growth.

### 2.4 Potentiodynamic polarization curve analysis

In the electrochemical experiments, the test piece was placed in an electrolyte of phosphate-buffered solution (PBS). An electrochemical workstation (Jiehan 5000, Taiwan) was used to obtain potentiodynamic polarization curves indicating the corrosion resistance (i.e., corrosion potential and corrosion current) of the material surface, while immersed in PBS. Polarization curves were measured with an onset potential of −3.0 V and a stop potential of +2.5 V at a scanning rate of 0.001 V/s. The counter electrode (CE) was a platinum plate. The reference electrode (RE) was a saturated calomel electrode (SCE). The working electrode (WE) was the sample being tested.

### 2.5 Cellular responses

The osteoblasts (MG63) used in this experiment were purchased from the Bioresource Collection and Research Center (BCRC 60279). The MG63 were cultured in Minimum Essential Medium (MEM, Gibico-Thermo Fisher Scientific) supplemented with 10% fetal bovine serum, 1.5 g/L sodium bicarbonate, and 1% penicillin/streptomycin and grown in an incubator (at 37°C and in 5% CO_2_). All cell experiments were performed at the School of Dentistry, Taipei Medical University. The front and back of the test pieces were sterilized using ultraviolet light on a sterile operating table. MG63 cells were then cultured on the surfaces of the test pieces at a concentration of 1.6 × 10^5^ for 3 h. Note that the area of the test piece was 1 cm^2^. After fixing the cells on the surface, the samples were subjected to alcohol gradient dehydration and critical point drying (CPD, LEICA EM CPD 030) to facilitate the observation of cell extension morphology, cell attachment, and cell-to-cell interactions. MG63 cell were cultured in DMEM containing 10% fetal bovine serum at 37°C.

### 2.6 Statistical analysis

Experiment data are expressed as the mean ± standard deviation (SD). All measurements were performed in triplicate. Three samples from each test group were measured at each time point. The Student’s *t*-test was used to analyze the effects of surface treatment on surface properties and cellular responses, with significant effects indicated by a *p*-value of < 0.05.

## 3 Results

### 3.1 Material surface properties

#### 3.1.1 Surface morphology and element analysis of materials following immersion in culture medium

**FIGURE 2:**
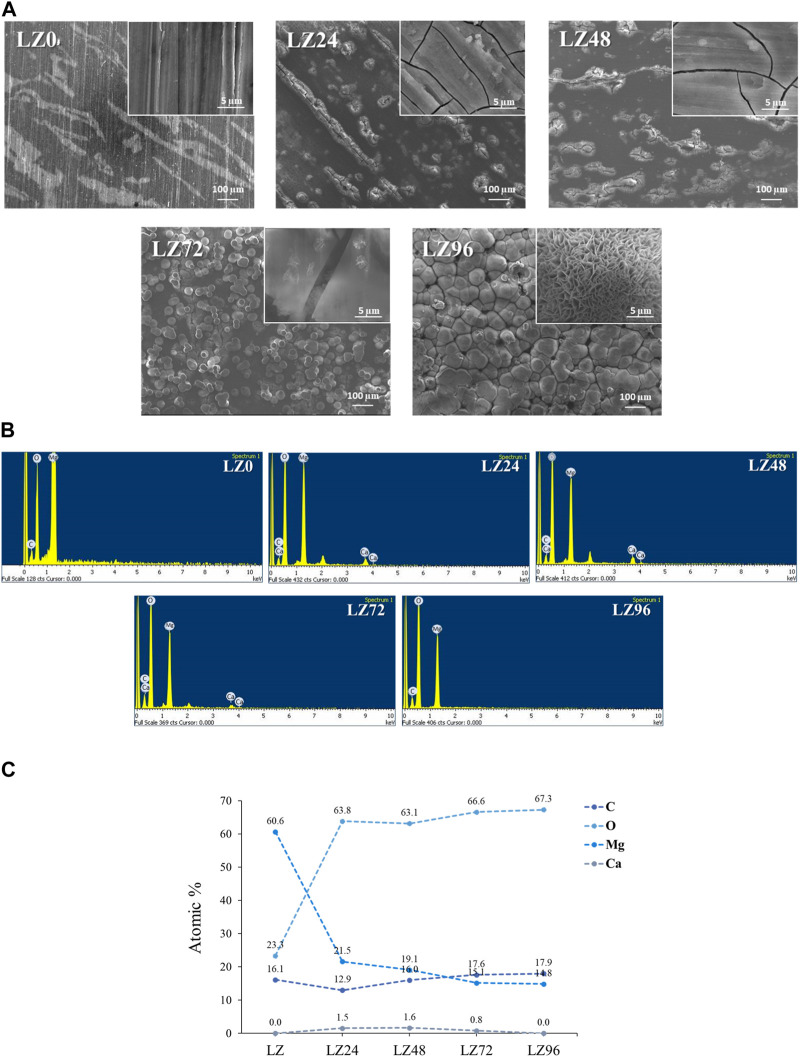
**(A)** FE-SEM results showing surface morphology of alloy samples; **(B)** EDX results showing the chemical composition of alloy samples; and **(C)** exact values of elements.

In this study, binary Mg-9wt.%Li alloy (LZ) was selected as a substrate. Note that the number following the LZ designation indicates the time spent immersed in culture medium at the time of testing (in hours). Optical microscopy and FE-SEM revealed that after immersion in culture solution for 0, 24, 48, 72 or 96 h, the LZ alloy exhibited deposits of corrosion products and a rough surface morphology (see Figure 2A). EDS analysis revealed that the initial corrosion deposits contained calcium from the cell culture solution; however, no calcium was observed in the deposits after 96 h of immersion, indicating that the Ca ions had been deposited with Mg (Figure 2B).

#### 3.1.2 Surface crystal composition following immersion in culture medium

**FIGURE 3:**
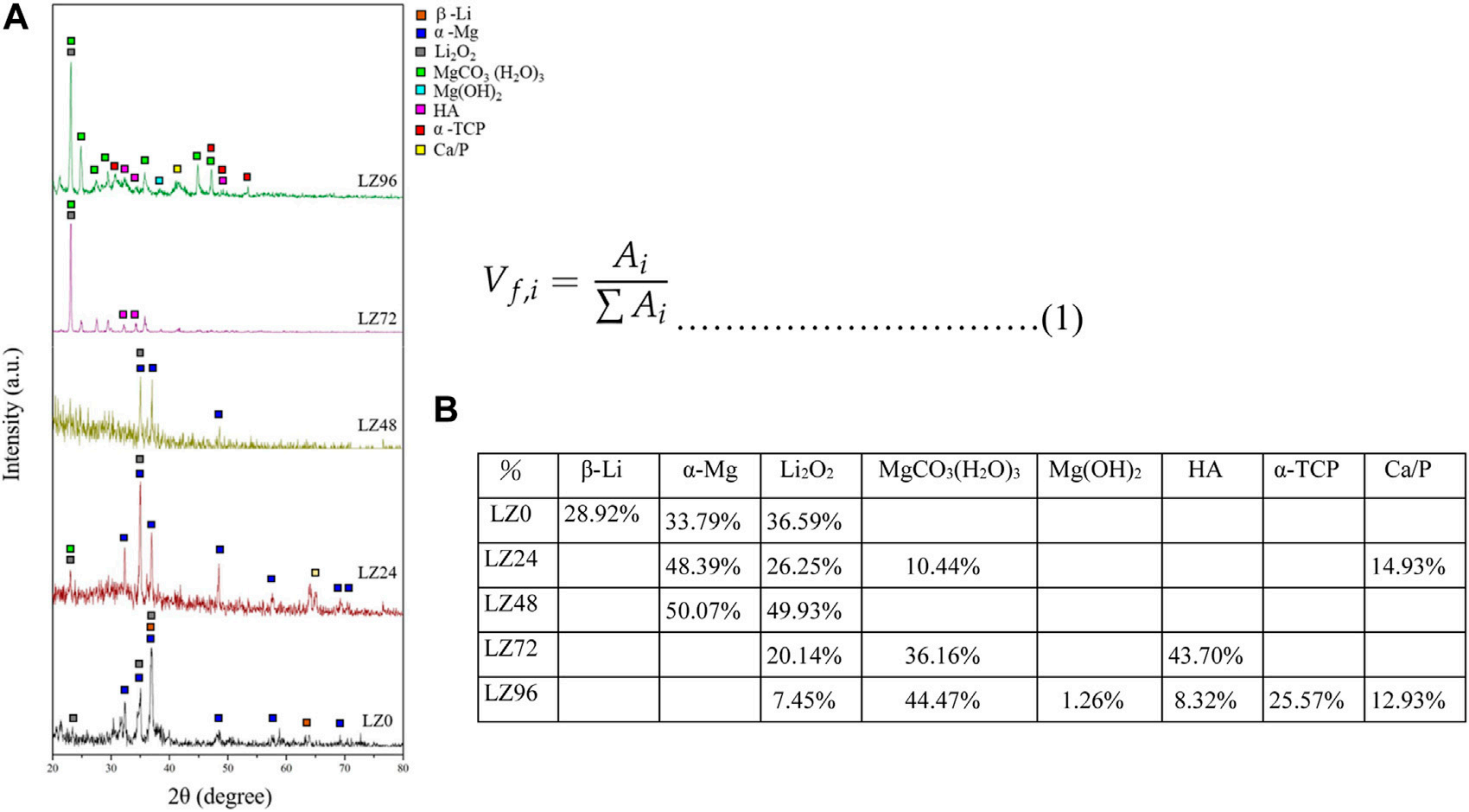
XRD results show the element composition on sample surfaces as a function of immersion time in the culture medium. **(A)** qualitative spectrum; **(B)** quantitative volume percent data.

As shown in Figure 3A, the Primary phase (main peak) is Mg and Li and the secondary phase is Li_2_O_2_. The surface of the untreated sample (LZ0) at room temperature presented two crystal phase structures composed of Mg or Li alloy products (β-Li and α-Mg). α-Mg was also detected on the surfaces of LZ24 and LZ48 samples. HA, and α-TCP crystal phases were detected on the surfaces of LZ72 and LZ96 samples. Li_2_O_2_ was detected in all samples. Li_2_O_2_, MgO_2_, hydroxides (Mg(OH)_2_) and carbonates (MgCO_3_ (H_2_O)_3_) were also detected on the surface of LZ72 and LZ96. The peak of LZ96 was more obvious than that of LZ72.

The three phases of β-L, α-Mg and Li_2_O_2_ tend to decrease with the increase of immersion treatment time, and even after 72 h of immersion, β-Li and α-Mg have not been detected at all. By analyzing the integrated peak area of each phase on the XRD pattern, the volume fraction of each phase in the coating can be calculated using the Pearson VII function in software such as Origin. Using this calculation, it is possible to determine the relative amounts of the different phases present in the coating and assess its overall composition (Amukarimi and Mozafari, 2021). The equation used is shown in Formula 1.

It is particularly worth noting that the Li oxide (Li_2_O_2_) tends to increase from 0, 24, and 48 h the longer the soaking time. It was further found that Li_2_O_2_ decreased as the immersion time increased to 72 h. And there are other products. For example, MgCO_3_ (H_2_O)_3_ and HA.

#### 3.1.3 Surface roughness following immersion in culture medium

**FIGURE 4:**
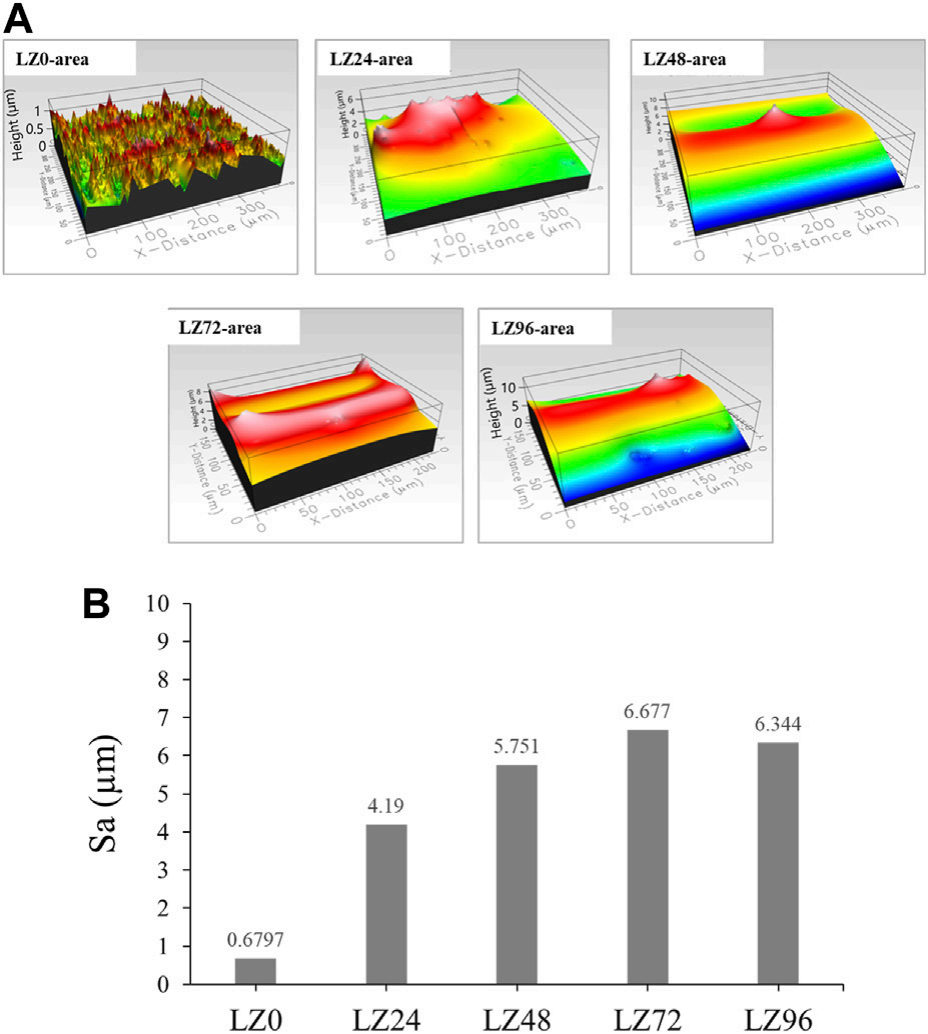
Roughness (Sa) of LZ0, LZ24, LZ48, LZ72, and LZ96 samples: **(A)** Surface morphology of all test samples; **(B)** Quantitative roughness results of all samples.

Surface roughness was shown to increase with immersion time. Immersion for 72 h resulted in a relatively flat surface profile. The surface roughness of samples LZ72 and LZ96 groups was very similar (Sa = 0.6, 4.2, 5.7, 6.7, and 6.3 μm) (Figures 4A, B). The surface roughness of L24 samples was 7-fold higher than that of the untreated group, resulting from flaky surface deposits of reaction products.

### 3.2 Corrosion resistance

#### 3.2.1 Analysis of corrosion resistance

**FIGURE 5:**
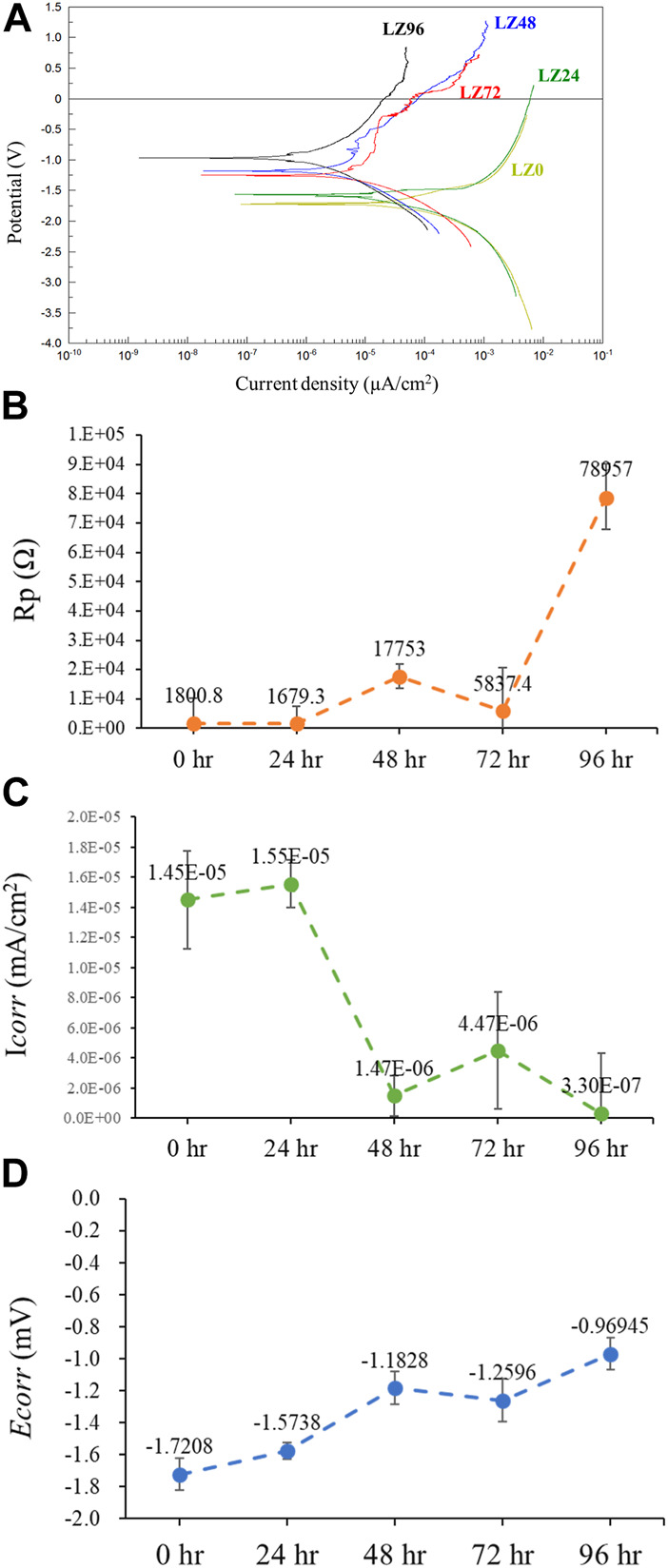
Electrochemical analysis of samples LZ0, LZ24, LZ48, LZ72, and LZ96 following immersion in PBS: **(A)** Potentiodynamic polarization curves; **(B)** Rp; **(C)** Icorr; and **(D)** Ecorr.

Potentiodynamic polarization (PD) curves were obtained by recording changes in potential, current values, and resistance using a potentiostat. This study used these values to assess the corrosion resistance of alloys immersed in PBS. PD tests were performed between a potential of −3.0 V (vs. OCP) and 2 V at a scanning rate of 0.1 mV s^−1^. Figure 5 presents the potentiodynamic polarization, polarization resistance (Rp), corrosion current values (Icorr), and corrosion potential (Ecorr) of the samples as a function of immersion duration. The fitting results of Ecorr, Icorr, and Rp were calculated by Faraday’s law using Eq. (15).

The Ecorr and Rp values of LZ96 samples exceeded those in the other groups, while the Icorr value of LZ96 was the lowest (at least 10-fold lower than that of the untreated control). Ecorr and Rp values increased and Icorr decreased with immersion duration. We also determined that the surface corrosion resistance improved with an increase in immersion duration. We speculate that the reaction with the cell culture medium resulted in the formation of a corrosion-resistant layer on the surface of the alloy.

#### 3.2.2 Ion release into the culture medium

**FIGURE 6:**
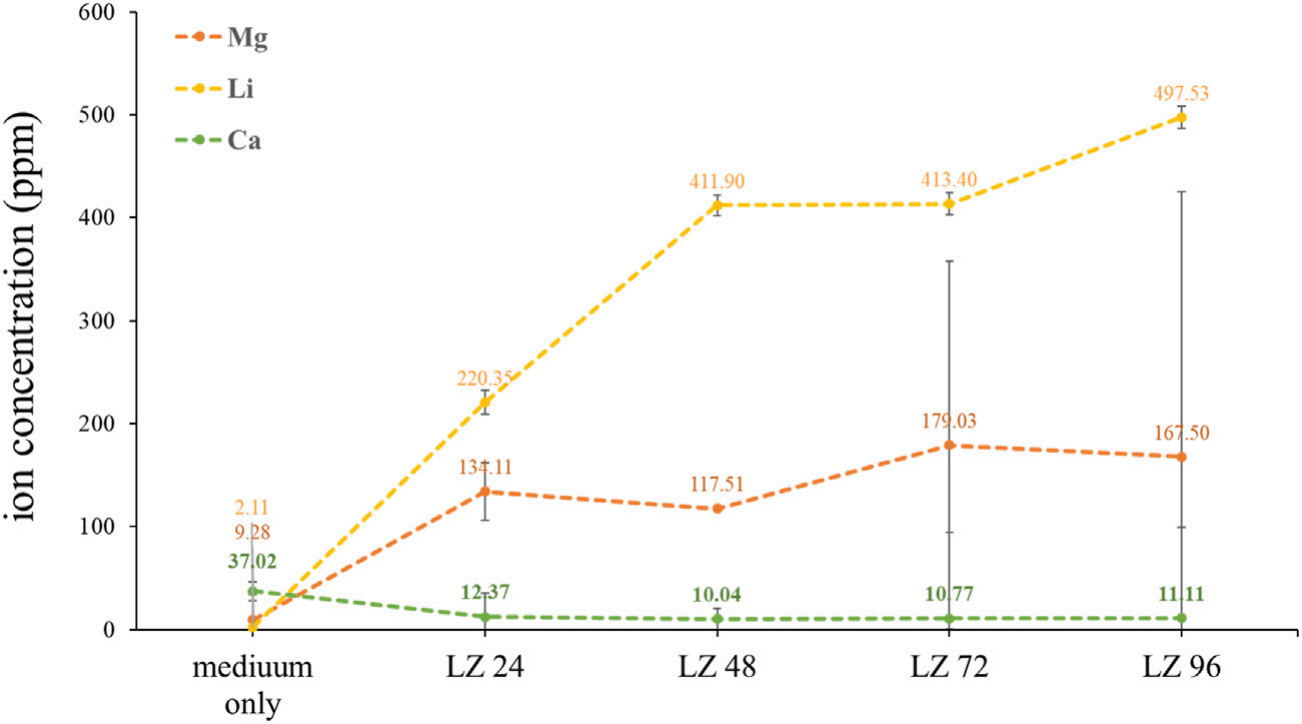
Li, Mg, and Ca ion concentrations in culture media following alloy immersion for 0, 24, 48, 72, or 96 h.

Samples of cell culture fluid collected at various time points throughout the immersion period were analyzed using ICP-OES to quantify the release of ions by the alloy into the culture medium. As shown in Figure 6, the concentration of lithium ions increased linearly with immersion time, as indicated by the Li^2+^ concentrations: LZ24 (220 ppm), LZ48 (411 ppm), LZ72 (413 ppm) LZ96 (497 ppm). Note however that after an initial sharp increase in Li-ion concentrations, the levels gradually slowed down at around 72 h before increasing again during the last 24 h. The Mg^2+^ concentrations were as follows: LZ24 (134 ppm), LZ72 (179 ppm), and LZ96 (167 ppm). The release of Mg ions increased until 48 h, at which point it leveled off and increased only gradually thereafter. Calcium ion concentrations were as follows: medium only (37 ppm), LZ24 (12 ppm), LZ48 (10 ppm), LZ72 (11 ppm), and LZ96 (11 ppm).

#### 3.2.3 pH values

**FIGURE 7:**
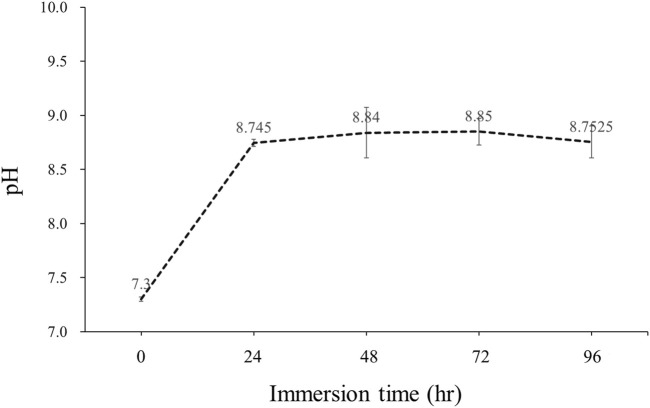
pH values as a function of Mg-Li alloy immersion time.

When immersed in an aqueous solution, the corrosion of magnesium alloy generates corrosion products and free species that can affect the pH of the solution. This experiment involved collecting cell culture medium to test the pH before immersion at various time points throughout the immersion period. The results were as follows: LZ0 (7.4), LZ24 (8.7), LZ48 (8.5), LZ72 (8.9), and LZ96 (8.7). Note that the pH of the magnesium alloy-impregnated solution increased rapidly in the first 24 h; however, pH levels leveled off thereafter (see Figure 7).

### 3.3 Cell attachment and initial cell morphology

**FIGURE 8:**
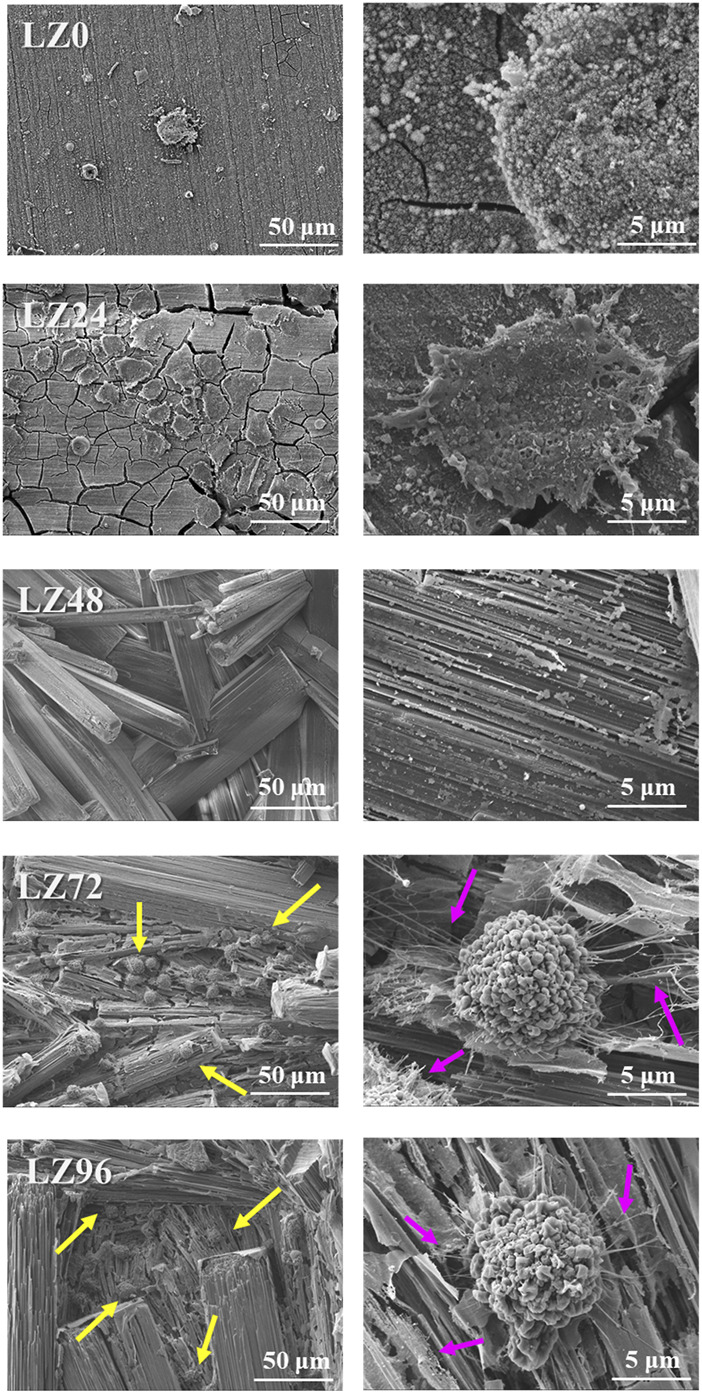
Adhesion morphology of MG63 cells on the surface of samples LZ0, LZ24, LZ48, LZ72, and LZ96. The yellow arrows indicate cells and the pink arrows indicate filopodia.

Cell adhesion is crucial to the survival of anchorage-dependent cells on biological matrices. In this study, cell adhesion was assessed by incubating MG63 cells on the surface of alloy samples for 3 h (Figure 8). FE-SEM was then used to observe cell adhesion, growth, and morphology. Almost no cells adhered to the surface of LZ0 control samples. A few cells adhered to sample LZ24; however, they were inactive. Cells readily adhered to samples LZ72 and LZ96 and appeared to be in a healthy state with a robust morphology. It appears that the long sheets of corrosion products on the surface of the samples presented good attachment points for pseudopodia. The yellow arrows in Figure 7 indicate cells attached to the alloy surface and the pink arrows indicate filopodia extensions. Note that the morphology of cells on LZ72 and LZ96 did not differ significantly.

## 4 Discussion

In pilot experiments, we observed no significant difference in the pH of the cell culture medium following immersion for 96, 120, 136, or 144 h (data not shown). We, therefore, selected 96 h as the longest immersion time in the current study. Corrosion resistance analysis and cell response tests were performed after immersion for 24, 48, 72, or 96 h.

### 4.1 Surface morphology and element analysis of alloys following immersion in culture medium

This study examined simple binary Mg-9wt.%Li alloys. Visual observation and FE-SEM analysis of the proposed LZ alloy revealed corrosion products and rough surface morphology. EDX analysis also revealed the presence of Ca on the surface of samples after immersion for 72 h. We speculate that the cell culture medium was the source of the Ca. Note that no Ca was observed on the surfaces of samples that were not immersed in the culture medium (LZ0) or on the surfaces of LZ96. It appears that corrosion progressed for roughly 72 h, at which point the corrosion products underwent redeposition on the samples. Sample LZ72 presented a few flake-like deposits of hydroxyapatite (HA), Ca-deficient HA, and β-TCP (Figure 2), whereas sample LZ96 was uniformly coated. The above-mentioned flakey deposits may account for the high roughness values obtained on the surface samples LZ72 and LZ96. Roughness increased linearly with immersion time until 72 h and then changed very little in the subsequent 24-h period (see Figure 2). Our results identified 96 h as the optimal soaking duration. The material is soaked in the culture solution for 96 h, which is the optimal soaking time for the experimental design confirmed by the data of this study. Electrochemical surface treatment is a relatively common surface treatment method, such as: micro-arc fluorination (Chen et al., 2021).

After grinding and polishing (i.e., before immersion), the Mg-Li alloy at room temperature exhibited a two-phase structure consisting of a Li-rich BCC structure β phase and a Mg-rich HCP structure α phase (Liu et al., 2109; Chang et al., 2006) (Figure 2). XRD results revealed that the surface compositions of LZ72 and LZ96 were identical. We selected samples covering a range of roughness values (Sa = 0.6, 4.2, 5.7, 6.7, and 6.3 μm) to analyze *in vitro* degradation as a function of immersion duration (Figure 3). The degree to which roughness changed (qualitatively) was also derived from data related to surface topography (see Figure 2).

### 4.2 Corrosion resistance

EDX analysis revealed a decrease in Mg ion concentrations at 96 h, due presumably to the redeposition of Mg ions released in the first 48 h (see Figure 3). Mg^2+^ plays a key role in bone development by promoting the attachment and differentiation of osteoblasts and accelerating mineralization to promote bone healing. During osseointegration, Mg^2+^ concentration also plays a key role in regulating bone regeneration (Cain and Labukas, 2020). Wang et al. demonstrated that Mg^2+^ (at a concentration of 10 mM) promoted the adhesion and proliferation of osteoblasts. Note however that at higher concentrations (>18 mM), Mg^2+^ significantly inhibited these effects (Cain and Labukas, 2020). The concentration of Mg ions in the culture medium (Figure 5) gradually increased with immersion time, with the ion concentration plateauing at 72 h and then gradually decreasing. Note that the ion concentrations were derived as follows: ppm = mM × MW, where MW indicates molecular weight. Mg^2+^ concentrations in the culture solution varied over time as follows: LZ24 (5.6 mM), LZ72 (7.3 mM), and LZ96 (7.0 mM).

This study was based on the hypothesis that the deposits that formed on the surface of LZ96 samples would protect against further corrosion. As for the underlying mechanism, we posit that the reaction of the Mg alloy with the cell culture medium led to the formation of a thick ceramic surface layer (possessing a crystalline structure), which blocked further corrosion (see Figure 7). This assertion is supported by the fact that the LZ sample surfaces were more stable than oxidized surfaces in cell culture media (Li_2_O_2_ layer) but less stable in water (Wang et al., 2014).

When Mg alloy implants were first assessed nearly a hundred years ago, it was found that the rapid degradation of the alloys led to the accumulation of large quantities of hydrogen gas in the form of subcutaneous bubbles, which essentially precluded the use of magnesium as a medical material (Wang et al., 2014). The primary chemical reaction involves the Mg and water (abundant in body fluids), wherein the formation of hydroxide ions and hydrogen gas involves the following chemical reaction:

$$Mg+2H_{2}O\;\to \;Mg\;{\left(OH\right)}_{2}+H_{2}$$ (1)

The corrosion of magnesium alloys generates corrosion products that can affect biocompatibility (e.g., hydrogen and hydroxide ions) (Tsakiris et al., 2021). For example, bubbles of hydrogen gas accumulating in the tissue surrounding implants can cause the separation of tissue layers, whereas hydroxide ions can cause surface alkalization sufficient to damage cells. Numerous researchers have sought to alloy Mg with nontoxic elements or coat the surfaces of Mg-based implants to counter these effects; however, our results indicate that this is not necessary as the rapid increase in alkalinity ceases within 24 h (Figure 5).

Limitations on sample preparation capacity made it impossible to fabricate the sample quantities required for conventional statistical analysis. Three samples were fabricated in each experiment run, and each run was repeated three times. Thus, the reported statistical values were derived from a total of nine samples, which should be sufficient to ensure data reliability (on par with previous papers). In future research, we will seek to develop techniques for the mass fabrication of samples.

### 4.3 Initial cell attachment and morphology

Cytotoxicity can be assessed in terms of cell response via direct or indirect testing (Yuan et al., 2019). In the current study, cell cultures were placed in direct contact with Mg alloy samples and then immersed in a cell culture medium. Following immersion in culture solution for 72 or 96 h, the Mg-Li alloy presented micron-scale spherical corrosion products. Culturing MG63 cells on samples LZ72 and LZ96 for 3 h was shown to cover pores at the micron to submicron scale. More importantly, cell culturing changed the structure of the corrosion products from a staggered lamellar structure into a long lamellar structure, corresponding to the three-dimensional porous fiber morphology of the extracellular matrix. Numerous studies have demonstrated that surfaces with a structure similar to that of the extracellular matrix are highly conducive to cell adhesion (Wang et al., 2017).

Surface deposits on Mg alloys can have a profound effect on cell-implant interactions and implant degradation (Johnson et al., 2012). The surface deposits that formed on Mg alloy implants after immersion for extended durations (72 or 96 h) were shown to facilitate cell adhesion and promote cell survival. Figure 8 presents a graphical abstract of this study. The immersion method used in this study was a simple inexpensive surface treatment process aimed at stimulating the natural physiological environment, leading to the formation of an anti-corrosion layer on the surface of the Mg alloy. The immersion solution used in this study was a cell culture with ion types and pH values very close to the physiological environment. As a result, the ions contained in the resulting sedimentary layer do not induce bio repulsion in the human body. This approach could likely be adopted for a wide range of applications.

One common disadvantage of magnesium alloys is their rapid degradation, which can compromise the implant support function before bone tissue healing is complete (Harrison et al., 2014). The degradation of magnesium alloys can lead to the generation of hydrogen ions (potentially causing emphysema) as well as hydroxide ions (generating a high pH microenvironment with negative effects on osteoblast growth and bone regeneration) (Kannan and Wallipa, 2013; Chen et al., 2014). Magnesium alloys also lack bioactivity and osteoconductivity (Hornberger et al., 2012; Razavi et al., 2014).

In the current study, magnesium alloys released ions at concentrations of 650–700 ppm (mg/L) (Figure 6), which is roughly 25 times the concentration required for normal physiology. Note that these concentrations were measured locally in the physiological environment with a reaction area of only 3 cm^2^. When compared with the size of the human body, it is reasonable to surmise that the release of magnesium ions at this concentration would not affect the normal physiological function of the overall organism.

**FIGURE 9:**
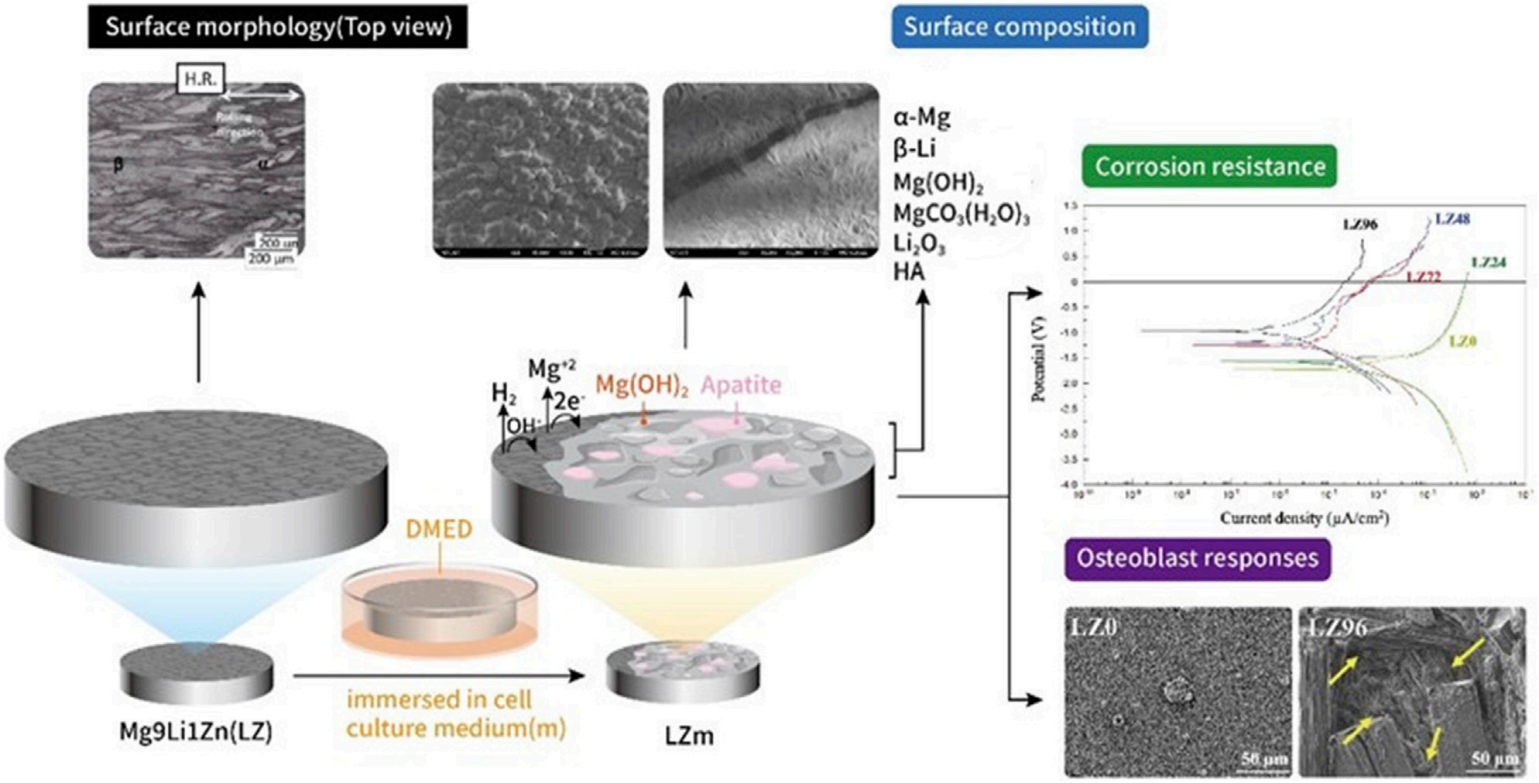
Schematic illustration showing that immersing Mg alloy in cell culture medium prompted surface degradation as well as the deposition of corrosion products, which slowed or halted further corrosion. The surface deposits included bioactive calcium and phosphorus compounds with a porous surface morphology (micron to nanometer scale), which was shown to promote cell adhesion and survival.

The surface treatment method proposed in this study was shown to slow the release of Mg ions to enable the gradual degradation of the material (Figure 9). Moreover, the degradation process fostered a microenvironment well-suited to the growth of osteocytes.

## 5 Conclusion

In this study, we sought to overcome the rapid degradation typical of Mg alloys when used as an implant material. This was achieved by preparing an Mg-Li alloy with a corrosion-resistant surface layer through the immersion of Mg alloy samples in a cell culture medium. The proposed surface treatment was shown to promote the formation of a surface coating, which enhanced the corrosion resistance of the Mg alloy and provided a microenvironment favorable for osteoblast attachment. The proposed surface modification scheme applies to the preparation of Mg alloy bone screws. The gradual degradation of the Mg alloy should delay the absorption of Mg ions by the human body, preserve the implant for a duration sufficient to ensure healing and eliminate the need for surgical resection, thereby avoiding the pain of secondary surgery. Note however that the effect of this surface treatment regimen on osseointegration will require further analysis. The proposed process is simple and easy to implement without the need for costly equipment.

## Data Availability

The original contributions presented in the study are included in the article/Supplementary Material, further inquiries can be directed to the corresponding author.
